# Pathogenesis, Diagnosis, and Management of Cytokine Release Syndrome in Patients with Cancer: Focus on Infectious Disease Considerations

**DOI:** 10.3390/curroncol32040198

**Published:** 2025-03-28

**Authors:** Panos Arvanitis, Andreas Tziotis, Spyridon Papadimatos, Dimitrios Farmakiotis

**Affiliations:** 1 Division of Infectious Diseases, The Warren Alpert Medical School of Brown University, Providence, RI 02903, USA; panagiotis_arvanitis@brown.edu; 2 Beth Israel Deaconess Medical Center Division of Gastroenterology, Boston, MA 02115, USA; atziotis@bidmc.harvard.edu (A.T.); spapadim@bidmc.harvard.edu (S.P.); 3 Beth Israel Deaconess Medical Center Division of Infectious Diseases, Boston, MA 02115, USA

**Keywords:** cytokine release syndrome, infectious complications, immunotherapy

## Abstract

**Background:** Cytokine Release Syndrome (CRS) is a hyperinflammatory state triggered by immune therapies like CAR T-cell therapy and bispecific T-cell engagers (BiTEs). Characterized by excessive cytokine release, CRS often mimics infectious and inflammatory conditions, complicating diagnosis and treatment. Immunosuppressive therapies used for CRS further elevate the risk of secondary infections. **Methods:** A systematic search of PubMed and EMBASE was conducted using terms related to “cytokine release syndrome”, “cytokine storm”, “infections”, and “management”. Studies were included if they described infectious complications, diagnostic mimics, or therapeutic approaches related to CRS. **Results:** Of 19,634 studies, 2572 abstracts were reviewed. Infections occurred in up to 23% of patients post-CAR T therapy and 24% post-BiTE therapy. Pathogens included gram-positive and gram-negative bacteria, herpesviruses (e.g., CMV, HSV), fungi (e.g., *Candida*, *Aspergillus*), and parasites (e.g., Toxoplasma gondii). CRS mimics also included non-infectious inflammatory syndromes. Differentiation remains challenging, but cytokine profiling and biomarkers (e.g., ferritin, CRP, sIL-2Rα) may aid in diagnosis. Treatments included tocilizumab, corticosteroids, and empiric antimicrobials. Prophylactic strategies were inconsistently reported. **Conclusions:** Effective CRS management requires early recognition, differentiation from infectious mimics, and collaboration between oncology and infectious disease (ID) specialists. A multidisciplinary, collaborative, and structured approach, including dedicated ID input and pre-treatment evaluation, is essential for optimizing CRS management and patient outcomes.

## 1. Introduction

Cytokine Release Syndrome (CRS) is a life-threatening systemic response characterized by the excessive release of pro-inflammatory cytokines [1]. Initially presenting with flu-like symptoms, CRS can rapidly escalate to severe organ dysfunction, posing a critical challenge in the management of immune-based cancer therapies [2,3]. The pathophysiology involves a complex cascade of immune activation, resulting in systemic inflammation and organ damage if untreated. This process can be misdiagnosed or mistaken for related conditions such as systemic inflammatory response syndrome (SIRS) from sepsis, macrophage activation syndrome (MAS), and drug-induced or other non-infectious causes of fever, all of which often occur concurrently in patients with cancer and share overlapping features [3,4,5,6].

CRS is widely recognized as an anticipated side-effect of cancer treatments such as (in order of frequency and severity) CAR T-cell therapy, Bispecific T-cell Engagers (BiTE) or antibodies, and immune checkpoint inhibitors [1,5,7,8] (Table 1). These therapies can also lead to immune effector cell-associated neurotoxicity syndrome (ICANS), a significant and often concurrent adverse effect, particularly in patients receiving CAR T-cell therapy [9]. The overlap between CRS and ICANS complicates diagnosis and management, as both syndromes can present with overlapping clinical features, necessitating vigilant neurological monitoring in affected patients [10].

Additionally, the associated immune suppression to counter the inflammatory response, as well as immune suppression from prior treatment lines, particularly with CAR T-cell therapy, amplifies patient vulnerability to secondary infections, which may mimic or exacerbate CRS and ICANS, creating diagnostic and therapeutic challenges [2,35]. Opportunistic infections (OI) caused by bacterial, fungal, and viral pathogens, including immune reconstitution syndrome (IRS), can closely mimic CRS, further complicating the management of patients, especially those already heavily immunosuppressed [35,36,37]. Those specific to the Central Nervous System (CNS), such as cryptococcosis, Human Herpesvirus-6 (HHV6) encephalitis, or Progressive Multifocal Leukoencephalopathy (PML), are important considerations in the differential diagnosis of ICANS [10,38].

Accurate diagnosis of CRS requires careful clinical assessment and biomarker evaluation, with emerging tools such as cytokine-based models showing promise in distinguishing CRS from sepsis and other infectious complications [5,39]. Antimicrobial therapy plays a crucial role in the empiric management of CRS, especially when infections co-occur. Empiric broad-spectrum antimicrobials are often necessary while awaiting more diagnostic information, but can often be promptly de-escalated, emphasizing the need for dedicated infection control and stewardship programs among patients with cancer [7,35]. Effective CRS management requires a multidisciplinary approach that integrates infectious disease (ID) expertise, oncology care, and advanced diagnostics to ensure favorable patient outcomes [2,7].

This review is, to our knowledge, the first effort to provide a comprehensive overview of the pathophysiology, clinical presentation, differential diagnosis, and management of CRS, focused on ID considerations and with practical recommendations for clinicians. Specifically, we highlight the challenges posed by infectious and other mimics of CRS, as well as the interplay between CRS-directed immunosuppression and resultant opportunistic infections.

## 2. Methodology

We conducted a systematic literature search in the PubMed and EMBASE databases to identify studies exploring ID considerations in the diagnosis and management of CRS in patients with cancer. The search was performed without restrictions on publication date and utilized a combination of keywords and Boolean operators. Key search terms included “Cytokine Release Syndrome (CRS)” OR “CRS Infections” OR “CRS Infectious Complications”, “CRS Cancer” OR “CRS Malignancy”, “Diagnosis” AND “Cytokine Release Syndrome”, “Biomarkers” AND “Infection” AND “CRS”, “Cytokine Storm” AND “Cancer”, and “Pathophysiology” AND “CRS” AND “Infections”. Additional terms incorporated into the search were “Antimicrobial Therapy” AND “Cancer” AND “CRS”, “CAR T-cell therapy” AND infection AND management, “IL-6 Blockade” OR “Tocilizumab” AND “Infections”, and “Febrile Neutropenia” AND “Cytokine Release Syndrome” AND cancer, among others. Last, we included search terms related to emerging immunotherapies associated with CRS, such as “Bispecific T-cell engager (BiTE) therapy” AND/OR “CRS” AND/OR “Infections”, “Bispecific Antibodies” AND/OR “CRS”, and “T-cell Redirecting Therapy” AND “Infectious Complications”.

The search results were screened for relevance based on titles and abstracts, and full texts of eligible articles were reviewed for inclusion. Studies were included if they addressed ID considerations related to CRS in patients with cancer, discussed diagnostic challenges or differential diagnoses, or provided insights into management strategies, including the use of biomarkers and antimicrobial therapies. Studies focusing solely on the pathogenesis of CRS without discussing infectious complications or providing insufficient clinical or diagnostic details were excluded.

## 3. Results

**Figure 1:**
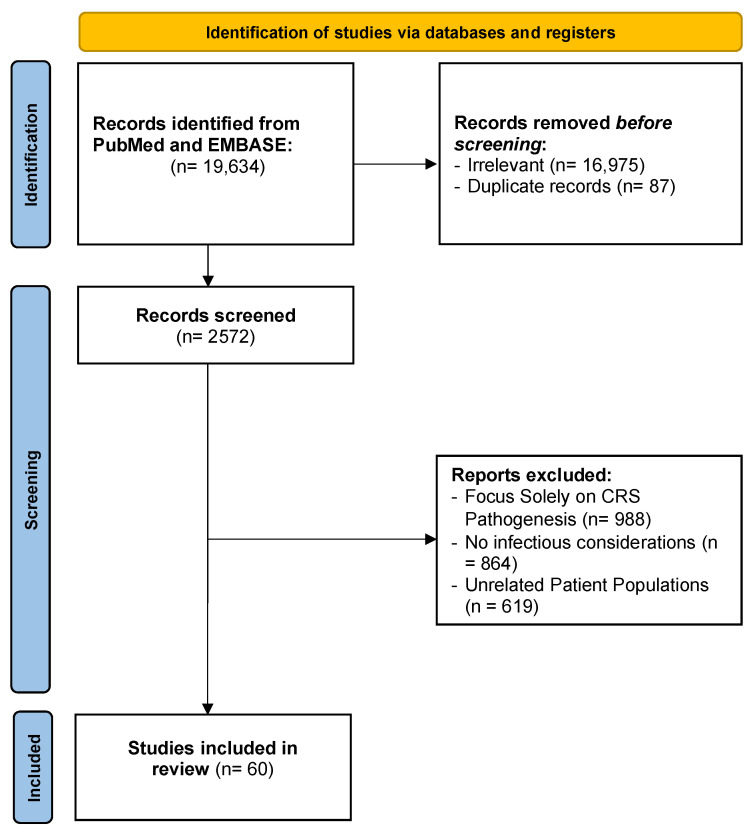
PRISMA Flow Diagram. CRS = Cytokine Release Syndrome.

Our systematic search identified 19,634 records from PubMed and EMBASE (Figure 1). Of these, literature on checkpoint inhibitors, IL-6 blockade, tocilizumab, febrile neutropenia, and CAR T therapy yielded 5573 records, with 1215 abstracts reviewed. Searches related to bispecific T-cell engager therapy, bispecific antibodies, and T-cell redirecting therapies retrieved 1377 records, with 177 abstracts reviewed. General searches on CRS and infectious complications identified 7069 records, with 235 abstracts reviewed. Additional searches on CRS diagnosis, biomarkers, cytokine storm, and pathophysiology resulted in 1635 records, with 488 abstracts reviewed. Searches focusing on CRS differential diagnoses, cancer, infection, and immunotherapy retrieved 2480 records, with 233 abstracts reviewed. Literature on CRS management, sepsis, and cancer produced 1500 records, with 224 abstracts reviewed. Additionally, 2572 records remained for screening. Of these reviewed abstracts, 988 studies were excluded for focusing solely on CRS pathogenesis, 864 for lacking infectious considerations, and 619 for involving unrelated patient populations. Ultimately, 60 were deemed pertinent and cited in the body of the manuscript (Figure 1).

### 3.1. Pathogenesis

**Figure 2:**
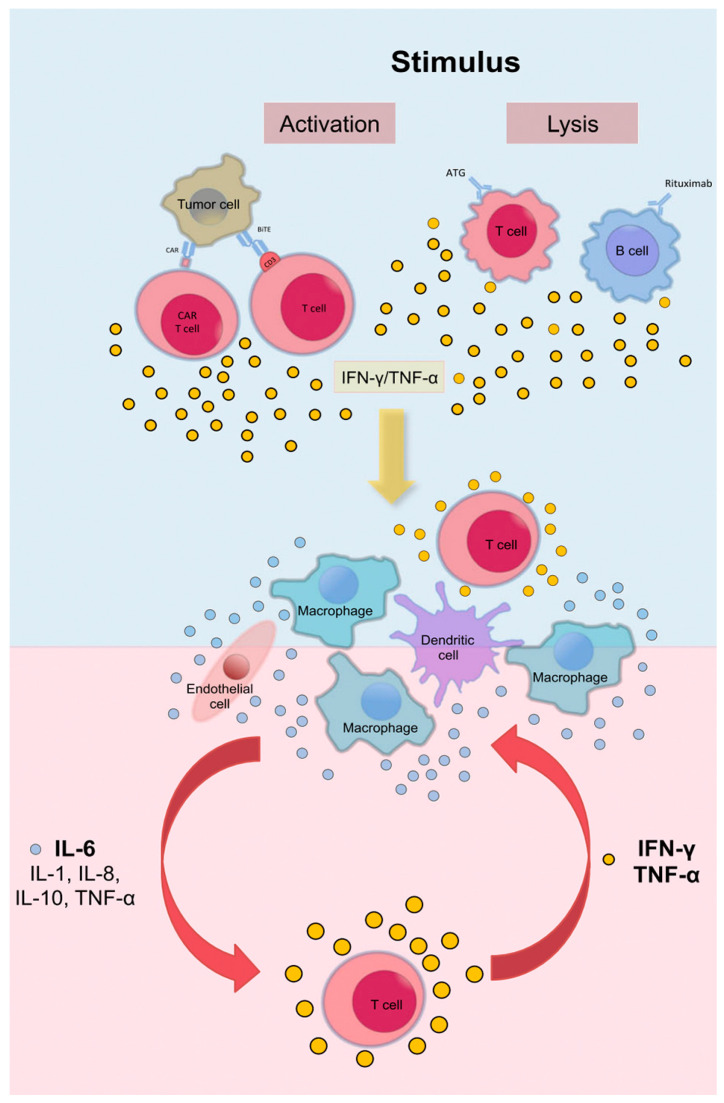
CRS can result from direct target cell lysis, releasing cytokines such as IFN-γ and TNF-α, or from therapeutic activation of T cells, leading to further cytokine release. These cytokines activate innate immune cells like macrophages and endothelial cells, triggering a cascade of inflammation and additional cytokine release. CAR: chimeric antigen receptor; IFN-γ: interferon-gamma; TCR: T cell receptor; TNF-α: tumor necrosis factor-alpha. Adapted from Shimabukuro-Vornhagen et al. [1], with permission.

The pathogenesis of CRS is based on a cascade of inflammatory events initiated by extensive immune cell activation. The process initiates with the simultaneous activation of multiple immune cell types, including T cells, B cells, natural killer (NK) cells, macrophages, and dendritic cells. This activation triggers the release of a diverse array of cytokines, with TNF-α serving as one of the earliest mediators [1,40]. During this initial phase, activated T cells additionally release IFN-γ, IL-2, IL-8, and MIP-1β, causing a widespread inflammatory response [40,41,42]. Macrophages play a central role in CRS by releasing significant amounts of IL-6 and IL-1, which drive a self-perpetuating feedback loop characterized by endothelial activation and vascular leakage, hallmark features of CRS [43,44,45]. IL-6 is a key mediator, contributing to fever, capillary leak syndrome, and hypotension [39,46]. Elevated levels of IFN-γ amplify macrophage activation, leading to the downstream release of TNF-α, which exacerbates tissue damage and systemic inflammation [41,43] (Figure 2). Of note, this excessive cytokine release can result in immune exhaustion, increasing vulnerability to secondary infections [47,48]. In severe cases, the activation of coagulation pathways further exacerbates microvascular injury, contributing to disseminated intravascular coagulation [46].

**Figure 3:**
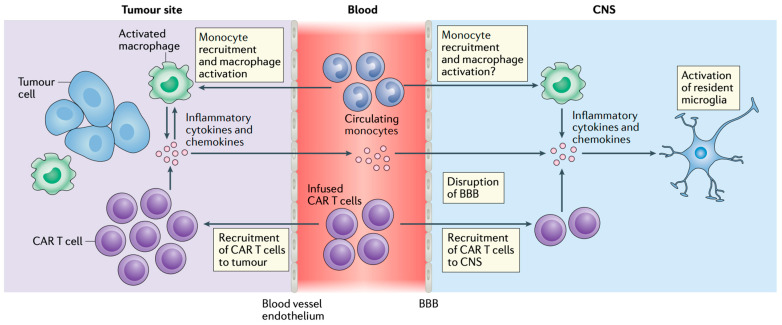
ICANS Pathophysiology: ICANS likely begins with CAR T-cells and activated immune cells, such as macrophages, releasing pro-inflammatory cytokines in the tumor microenvironment. These cytokines—including IL-1β, IL-6, IL-10, CXCL8, CCL2, IFN-γ, GM-CSF, and TNF—enter the bloodstream, disrupt the blood–brain barrier, and lead to cytokine and CAR T-cell accumulation in the CNS, activating resident microglia. Arrows represent directional flow or recruitment of cells and signaling molecules. Reproduced from Morris et al., 2022 [51], with permission.

ICANS shares this cytokine-driven pathophysiology, with blood-brain barrier disruption contributing to the penetration of inflammatory mediators into the CNS, resulting in neuroinflammation, cerebral edema, and neuronal dysfunction [49] (Figure 3). In severe cases of ICANS, the inflammatory cascade leads to profound neurotoxicity through endothelial activation and increased permeability of the blood-brain barrier [50]. The resulting neuroinflammation could potentially predispose patients to CNS infections, including cryptococcal meningoencephalitis and viral reactivations such as HHV6 encephalitis or progressive multifocal leukoencephalopathy (PML, JC virus reactivation) [38,50].

The severity of CRS is influenced by tumor burden and the specific CAR construct [41,45,52]. Tumor burden has consistently been identified as a strong predictor of severe CRS, with higher tumor loads significantly increasing the risk across various hematological malignancies [41,53]. For instance, in acute lymphoblastic leukemia (ALL), the number of lymphoblasts in the bone marrow prior to treatment directly correlates with CRS severity [43,44]. Similarly, patients with multiple myeloma who present with a high bone marrow tumor burden experience elevated rates of CRS [42]. In CAR-T-mediated CRS, CAR T-cells with CD28 costimulatory domains are linked to earlier onset and higher CRS rates due to their lower activation threshold [52]. Additionally, the choice of target antigen affects toxicity profiles; BCMA-directed CAR T-cells are associated with lower CRS rates compared to CD19-targeting constructs [43]. Interestingly, BCMA-directed bispecific antibodies, primarily used to treat multiple myeloma, are also the main agents in this class that have been associated with OI [54].

CAR-T-mediated therapies report a pooled severe CRS prevalence of only 13%, although disease stage has been identified as an independent risk factor for CRS severity [55]. BiTEs have demonstrated a high incidence of CRS in MM, with teclistamab causing CRS in 72.1% of treated patients, while baseline characteristics such as tumor burden do not clearly predict CRS severity [25]. In ALL, CD19-targeting BiTEs have a similarly high CRS incidence, reported at 78% [56]. Comparatively, patients with lymphoma treated with BiTEs exhibit lower CRS incidence and severity than those with ALL or MM [55].

Regarding ICANS severity, several patient-specific and biological factors contribute to its progression. Higher disease burden, pre-existing neurologic comorbidities, and a higher CAR T-cell dose have been identified as key risk factors [57]. Similarly to CRS, CAR T-cells construct characteristics influence neurotoxicity risk, with CD19-directed CAR T-cells being associated with more severe ICANS compared to BCMA-targeting constructs [57,58,59]. A high baseline inflammatory state further correlates with increased ICANS severity, suggesting that systemic inflammation before CAR T-cell infusion plays a crucial role in neurotoxicity risk [58]. Additionally, pre-infusion immune characteristics, such as a lower percentage of CD3CD8 lymphocytes and higher levels of monocytic-myeloid-derived suppressor cells, are associated with early neuroaxonal injury [58]. Neurofilament light chain (NfL) levels have emerged as an important biomarker, with elevated pre-treatment NfL levels serving as an indicator of pre-existing neuroaxonal injury and a predictor of both ICANS onset and severity [58]. Hypophosphatemia has also been identified as a predictor of ICANS severity, with lower levels correlating with earlier onset and worse neurotoxicity [60].

### 3.2. Clinical Presentation

**Figure 4:**
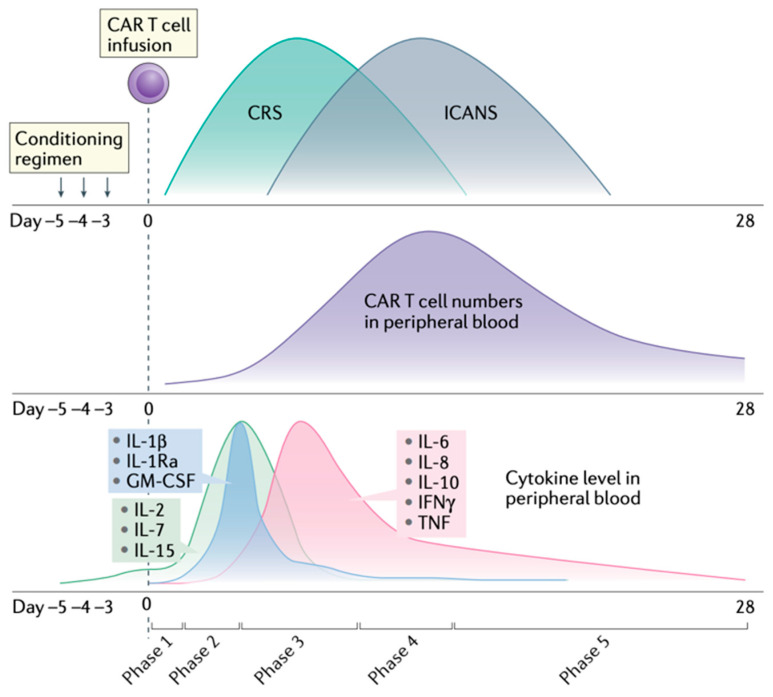
Schematic representation illustrating the relative timeline of CRS and ICANS, along with the kinetics of CAR T-cell expansion and cytokine dynamics in peripheral blood. Reproduced from Morris et al., 2022 [51], with permission.

The onset of CRS symptoms generally occurs within 1–14 days following the initiation of immunotherapy, although timing can vary depending on the therapeutic modality and individual patient characteristics [1,4,6,36,40,61,62] (Figure 4). Infections were observed in approximately 23% of patients within the first 30 days following CAR T-cell therapy, with higher rates among those experiencing severe CRS [63,64]. Similarly, bispecific T-cell engagers have been associated with infection rates up to 24%, further supporting the need to consider infectious complications in the differential diagnosis of CRS [63]. Unlike CAR T-cell therapy, CRS associated with bispecific antibodies often occurs earlier, frequently within the first 24 h of administration, necessitating distinct diagnostic and therapeutic approaches [16,65]. BiTE-related CRS can lead to endothelial dysfunction and increased vascular permeability, which may predispose patients to secondary infections by facilitating microbial translocation [53]. Furthermore, the continuous infusion required for BiTE therapy can increase the risk of catheter-associated bloodstream infections, further complicating the clinical picture [64].

CRS encompasses a broad spectrum of clinical manifestations, ranging from mild constitutional symptoms to severe, life-threatening organ dysfunction. These two extremes mimic mainly (early) viral (including respiratory viruses and Cytomegalovirus (CMV)) infections, and bacterial sepsis or severe IRS, respectively. For ICANS, in particular, besides encephalopathy of sepsis, the broad differential for CNS infection includes common but also opportunistic meningoencephalitis syndromes, including those by pathogens that can cause (“unmasking”) IRS, such as *Cryptococcus* and JC virus, the culprit for PML [10].

Early presentations typically include fever and fatigue, which may progress to hypotension, hypoxia, and tachycardia [1,9,40,52,62]. With such symptoms, it is important to rule out bacterial pneumonia and sepsis or a potentially hospital-acquired viral respiratory infection, usually by means of a multiplex PCR in a nasopharyngeal sample.

The severity of CRS is stratified using various grading systems, with the most recent and widely adopted being the American Society for Transplantation and Cellular Therapy (ASTCT) CRS consensus grading [62], which has replaced earlier frameworks [66]. The ASTCT system provides a standardized approach, categorizing CRS into four grades: from mild fever without hypotension or hypoxia (Grade 1) to life-threatening symptoms requiring vasopressors and mechanical ventilation (Grade 4). Advanced cases often involve hepatic dysfunction, marked by elevated liver enzymes, and acute kidney injury [4,6,62].

CRS frequently coexists with ICANS, presenting with confusion, ataxia, seizures, and, in severe cases, persistent headaches, nausea, vomiting, and altered mental status in the setting of cerebral edema [67]. In such cases, CNS infection may need to be ruled out, especially if the patient has been off anti-herpetic or cryptococcal (azole) prophylaxis.

Laboratory findings play a pivotal role in monitoring CRS progression and guiding management [39,43,44]. Elevated cytokine levels, particularly IL-6, IFN-γ, and TNF-α, are hallmark features of CRS and serve as diagnostic and prognostic biomarkers [7] (Figure 4). Other key markers include elevated inflammatory indicators such as CRP and ferritin, which reflect systemic inflammation and immune hyperactivation [44]. Cytopenias, including anemia, leukopenia, and thrombocytopenia, are frequently observed due to bone marrow suppression or immune-mediated destruction. Rarely, Parvovirus B19 infection can present in a similar way, with aplastic anemia or pure red blood cell aplasia, especially in patients with hypogammaglobulinemia, a common adverse event from CAR T cell, especially anti-BCMA, treatments [68]. Coagulation abnormalities, such as prolonged PT, elevated D-dimer levels, and reduced fibrinogen, indicate activation of the coagulation cascade and potential progression to disseminated intravascular coagulation, which can also be part of severe bacterial *Candida* (usually in the absence of antifungal prophylaxis) sepsis [44].

### 3.3. Differential Diagnosis

CRS poses significant diagnostic challenges due to its clinical similarities with a range of mostly infectious but other inflammatory conditions, as well, complicating both its identification and treatment. Conditions like sepsis, non-infection-related SIRS, HLH, and MAS share overlapping symptoms and signs, such as fever, hypotension, and organ dysfunction [50,52]. Additionally, bacterial, fungal, viral, and parasitic infections can mimic CRS, further complicating the differential diagnosis [1,10,69] (Table 2).

**Figure 5:**
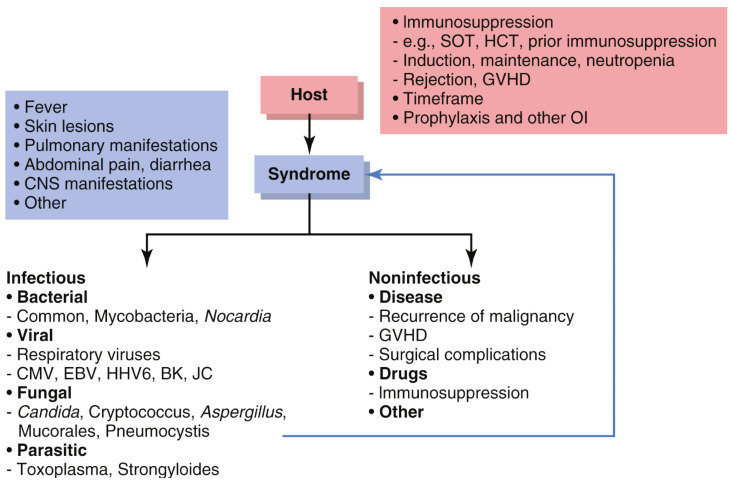
Structured approach to the diagnosis of CRS and other potentially infectious syndromes in the immunocompromised host. Reprinted with permission from Farmakiotis and Rogers in Wing et al. [89].

A structured approach for such complex patients is mandated and best summarized in Figure 5. First, it is important to understand the host, i.e., their timing, level, and type of immunosuppression, i.e., prior and current treatments, comorbidities, and prophylactic antimicrobials; second, identify the syndrome(s) (fever, pneumonia, skin lesions, CNS disease, abdominal symptoms); third, build a structured differential diagnosis of infectious vs. non-infectious causes. The authors of this article favor a kingdom-based approach for infectious causes, and specifically for addressing possible medication toxicities as well as underlying malignancy-related factors (Figure 5 and Table 2) [89].

SIRS presents, by definition, with nonspecific manifestations: fever, tachycardia, tachypnea, and leukocytosis or leukopenia, which overlap with the early manifestations of CRS. These signs reflect a systemic response to diverse stressors, mainly severe infections (sepsis), as well as non-infectious causes (such as pancreatitis, organ infarction, or trauma), rather than the immune-targeted activation seen in CRS. All patients with suspected CRS should have at least two sets of blood cultures and other syndrome-directed (e.g., urine cultures, imaging) work-up for infection (Table 2).

Certain biomarkers may help determine the role of infection in patients with symptoms and signs suspicious for CRS. A key distinction may be the absence of profoundly elevated cytokine (mainly IL-6 and interferon-gamma) levels in sepsis, which are distinguishing features of the cytokine storm in CRS, particularly in the appropriate clinical context [90]. Classic “inflammatory markers” like sedimentation rate and CRP are of little to no use, since they are markedly elevated in almost all inflammatory syndromes. However, novel biomarkers for bacterial or fungal sepsis, such as procalcitonin or adrenomedullin have shown promising sensitivity and specificity in identifying infection, but, to our knowledge, have not been studied specifically in the context of CRS, yet [91,92].

Bacterial sepsis shares defining signs with CRS, including fever, hypotension, and tachycardia. However, it is often accompanied by distinct features such as positive blood cultures or organ-specific symptoms and signs [7,36]. Management strategies for sepsis differ significantly from those for CRS, necessitating targeted antimicrobial therapy.

Secondary infections are common in patients receiving CAR T-cell therapy due to severe cytopenias, damaged mucosal barriers, and low immunoglobulin levels [9,46]. The latter seem to be more important for intermediate and long-term infection risk. Neutropenia, which will always happen early in CAR T-cell therapy, increases the risk of bacterial infections, including gram-negative and gram-positive bacteria, of which Pseudomonas is the most virulent. These infections often present with fever and hemodynamic instability, mimicking CRS and complicating diagnosis. The use of antimicrobial prophylaxis with an oral fluoroquinolone seems to be protective against severe infections and is routinely used at our center.

Uncommon infections such as tuberculosis, endemic mycoses (histoplasmosis, blastomycosis, coccidioidomycosis, paracoccidioidomycosis), and parasitic infections can closely mimic CRS in patients with specific exposure histories, complicating diagnosis [53]. Tuberculosis, particularly in its disseminated form, can present with prolonged fever, weight loss, lymphadenopathy, and multiorgan involvement, which may overlap with the systemic inflammatory features of CRS. Radiographic findings such as miliary patterns on chest imaging and positive interferon-gamma release assays or cultures will distinguish tuberculosis from CRS [6]. At our (and most similar) institution, all patients prior to CAR T-cell treatments are tested for latent tuberculosis infection with an IFN-g release assay and careful risk stratification by an ID specialist.

Endemic mycoses, such as disseminated histoplasmosis or blastomycosis, are another consideration, especially in patients with travel history or residency in endemic areas, such as the Grand Lakes in the US. These infections may manifest with fever, pancytopenia, elevated inflammatory markers, and hepatosplenomegaly, features that overlap with CRS [93]. Coccidioidomycosis is endemic in the South-West US (Arizona, Nevada, N. Mexico, W. Texas, and S. California, especially in the San Joaquim Valley area, hence the name “valley fever”) and in Central America. Laboratory confirmation of such mycoses typically requires antigen testing, fungal cultures, histopathology, or PCR [6]. Positive serologies can reflect past or active infection and are often used in the pre-CAR T or BiTE evaluation for risk stratification and the need for dedicated prophylaxis. Imaging studies may reveal multi-focal pneumonia, diffuse lymphadenopathy, or splenomegaly, aiding in differentiation from CRS [93].

In immunocompromised patients, particularly those who are not receiving trimethoprim/sulfamethoxazole prophylaxis (usually due to fear of side effects, especially myelosuppression, or sulfa allergy), *Nocardia* infections and even associated IRIS can mimic CRS due to overlapping systemic inflammatory responses, including fever, lung nodules, and brain abscesses [71]. Diagnosis relies on microbiological culture, PCR, or histopathology.

Parasitic infections, particularly strongyloidiasis, are a rare, but potentially critical mimic in immunocompromised patients, especially those receiving corticosteroids or T-cell immunosuppressive therapy. Strongyloidiasis can progress to hyperinfection syndrome, characterized by fever, gastrointestinal symptoms such as diarrhea and abdominal pain, respiratory distress, and multi-organ failure, which may be mistaken for severe CRS. Diagnosis requires stool ova and parasite testing, serologic assays, or molecular testing to confirm *Strongyloides* infection [94].

Opportunistic fungal infections such as those caused by *Aspergillus* and *Candida* species can also affect CAR T-cell recipients. Invasive aspergillosis may present with fever, respiratory symptoms, and nodular infiltrates on imaging, which overlap with pulmonary manifestations of CRS. Diagnostic confirmation often requires galactomannan antigen, beta-D-glucan (Fungitel^®^) testing, or fungal cultures. *Candida* infections, particularly in mucosal or disseminated forms, further complicate the clinical picture, as they can exacerbate systemic inflammation and organ dysfunction. Viral reactivations are another major concern, with CMV and herpes simplex virus (HSV) being the most common. CMV reactivation can lead to end-organ damage, including colitis, pneumonitis, or retinitis, and is associated with high levels of circulating inflammatory cytokines that mimic CRS. PCR-based viral load testing is crucial for differentiating CMV infection from CRS [36]. HSV reactivations, often presenting as painful mucocutaneous lesions or systemic symptoms, require high clinical suspicion and early antiviral therapy. The use of (val)acyclovir prophylaxis is very effective. Distinguishing secondary infections from CRS exacerbations can be challenging due to overlapping clinical signs, including fever, hypoxia, and hypotension. Diagnostic approaches incorporate microbiological testing, imaging, and cytokine profiling to delineate infectious from non-infectious etiologies [36]. Treatment strategies should include tailored antifungal or antiviral regimens when infections are confirmed [95].

MAS and HLH are hyperinflammatory syndromes that share clinical features with CRS, such as fever, hyperferritinemia, cytopenias, and multiorgan dysfunction. However, MAS and HLH can be differentiated by laboratory findings, including hypofibrinogenemia, elevated triglycerides, and hemophagocytosis on bone marrow biopsy, which are not present in CRS. MAS is often associated with autoimmune diseases, while HLH commonly arises due to (mostly viral) infections, malignancies, or genetic predisposition [95,96]. Serum ferritin levels, while elevated in all three conditions, typically reach significantly higher levels in MAS and HLH, often exceeding 5000 µg/L in MAS and 10,000 µg/L in HLH, whereas CRS tends to present with comparatively lower ferritin levels, typically less than 1500 µg/L [97,98,99,100]. Elevated soluble IL-2 receptor levels and coagulopathy are also more characteristic of HLH. MAS and HLH require immunosuppressive therapies such as corticosteroids or anakinra without the need for cytokine-modulating agents [90].

Drug-induced fever is an important noninfectious mimic of CRS that must be considered, particularly in patients receiving antibiotics, granulocyte colony-stimulating factors, or immune-modulating therapies [6]. Unlike CRS and sepsis, drug-induced fevers are almost never accompanied by hemodynamic instability, significant elevations in inflammatory cytokines, or end-organ dysfunction [6]. Such fevers typically resolve upon discontinuation of the causative agent [6].

### 3.4. Treatment and Prophylaxis

The management of CRS is based on the ASTCT criteria [62]. Grade 1 CRS typically responds to supportive measures, including antipyretics and intravenous fluid support. For Grade 2 and above, treatment focuses on immunotherapy, mainly using the IL-6 inhibitor tocilizumab, which has been highly effective in controlling severe CRS [62,66]. In cases of refractory CRS or Grade 4 disease, corticosteroids, particularly dexamethasone, serve as second-line agents, often used in combination with tocilizumab [2,101,102].

Early intervention with emerging therapies has shown promise in mitigating CRS and reducing its severity. For instance, Anakinra (IL-1 antagonist) has shown promise in treating severe CRS without interfering with CAR T-cell function, which has been demonstrated in several clinical studies [9]. Similarly, IFNγ-blocking agents like emapalumab and JAK inhibitors like ruxolitinib are being explored for their potential in managing refractory CRS [40,103]. These agents, while not yet widely implemented, offer new options to target the complex cytokine activity driving CRS [9].

Early recognition of CRS is crucial for optimizing outcomes, as the syndrome can rapidly progress from mild symptoms to life-threatening complications. Comprehensive assessments, including advanced imaging and repeated cytokine profiling, are essential to differentiate CRS from other clinical entities and to guide therapeutic interventions [6,36].

Prophylactic strategies in the use of CAR T therapies have evolved to include fractionated CAR T-cell dosing and preemptive tocilizumab administration [102]. Anakinra has also been proposed for prophylaxis in high-risk patients, showing potential in reducing both the frequency and severity of CRS episodes [9]. Additionally, granulocyte-macrophage colony-stimulating factor has shown promise in recent reports [3]. The timing of CAR T-cell infusion has also become a critical consideration, with recommendations to delay treatment in patients with active infections to minimize the risk of exacerbating both CRS and infectious complications [40]. In addition to these preventive strategies, infection risk following CAR T-cell therapy is influenced by a combination of disease-related, treatment-related, and host-related factors, reflecting the multifactorial nature of infection risk in patients receiving cellular immunotherapies. Patients with ALL, multiple prior lines of therapy for MM, or those receiving higher CAR T-cell doses have shown increased susceptibility to infections [53,104]. Severe CRS, which is commonly treated with corticosteroids or tocilizumab, may further increase infection risk due to the immunosuppressive effects of these therapies [53]. Persistent hypogammaglobulinemia due to B-cell or plasma cell depletion, especially following BCMA-targeted therapies, contributes to delayed respiratory infections and impaired long-term immune reconstitution [53,105]. Additionally, BiTEs are associated with significant cytopenias and immune dysfunction, with reported infection rates comparable to those seen with CAR T-cell therapy [54].

Indeed, the management of CRS is further complicated by the heightened risk of infectious complications, driven by immune dysregulation and treatment-related immunosuppression. This requires a proactive approach to infection prevention and management, including the use of empirical broad-spectrum antibiotic coverage in febrile patients [40]. Antibacterial (levofloxacin) and antifungal (fluconazole or even mold-active triazoles: voriconazole, posaconazole, isavuconazole) agents during the duration of neutropenia, as well as anti-*Pneumocystis* and antiviral prophylaxis agents the herpesviruses, have become standard practice during the high-risk period following CAR T-cell infusion [40]. Trimethoprim/sulfamethoxazole prophylactic use after CAR T-cell and during BiTE treatments likely also has broader antibacterial activity, including *Nocardia*. Serial CMV DNA measurements can help diagnose and treat reactivation early, preventing CMV disease and antiviral toxicity (mainly cytopenias from valganciclovir).

The role of the ID consultant is key in preventing serious complications among patients experiencing or at risk for CRS. At our institution, all patients undergo thorough ID evaluation prior to CAR T-cell therapy initiation, which serves the following main purposes: 1. To evaluate for possible active infection that could complicate treatment; 2. to identify and treat latent infections, such as latent tuberculosis, endemic mycoses, or Strongyloidiasis; 3. to oversee vaccinations and other preventative measures, including lifestyle counseling (such as avoidance of well water) peri- or post-CAT-cell treatment; 4. to establish a patient–ID provider relationship, given the ongoing risk for severe and complex infections in this patient population.

## 4. Conclusions/Future Directions

While major advances have been made in understanding the pathophysiology and treatment of CRS, challenges remain in balancing the efficacy of immunotherapy with the risk of infectious complications. Current therapeutics, such as tocilizumab and corticosteroids, have proven effective^9^. Emerging therapies include anakinra, emapalumab, and JAK inhibitors, which seem to be promising alternatives for refractory cases and prophylaxis [9,102,103].

Future efforts should prioritize the development of more targeted, less toxic therapies. Advancements in biomarker discovery and cytokine profiling hold potential for improving the early diagnosis and grading of CRS, and more importantly, differentiating it from sepsis and other mimics [6,36].

Strategies to reduce infectious complications without selecting for resistance are essential, particularly in the contexts of CAR T-cell therapy and BiTE. Enhanced infection prevention protocols, such as the use of pathogen-specific prophylaxis and novel antimicrobial agents, are necessary to mitigate the risk for antibacterial and antifungal resistance in immunosuppressed patients [40].

Another area of interest is CAR T-cell design, including the use of next-generation CAR constructs and dosing regimens that minimize cytokine release while maintaining efficacy. The further refinement of switchable CAR systems and fractionated dosing strategies could significantly reduce the incidence and severity of CRS [3]. Additionally, clinical trials exploring therapies targeting more downstream cytokines could further expand the therapeutic arsenal and improve patient outcomes. However, our experience has shown that sometimes, off-target effects of novel immunomodulators can lead to unexpected susceptibility to opportunistic infections, for which active surveillance is advised.

Efforts to mitigate ICANS have focused on targeting key inflammatory pathways. Tocilizumab, while widely used for CRS, does not cross the blood–brain barrier and has limited utility in ICANS. Agents such as anakinra have been proposed for neurotoxicity management [53,104]. To address broader toxicities, including on-target off-tumor effects, novel CAR T-cell platforms like the Peri Cruiser CAR-T have been developed, offering promising strategies for enhancing CAR T-cell therapy safety across broader indications. This system incorporates miRNA cassettes targeting adhesion molecules required for T-cell extravasation into healthy tissues. In preclinical models, this design reduced infiltration into normal organs and prevented systemic toxicity without compromising antitumor activity [106].

In conclusion, while significant progress has been made, CRS remains a complex and challenging entity for multi-disciplinary hematology–oncology teams. Continued research into the underlying mechanisms, diagnostic tools, and innovative treatments is crucial for advancing the field and ensuring that the benefits of immunotherapy are maximized while minimizing associated risks. Multidisciplinary collaboration among oncologists, immunologists, ID specialists, and basic science investigators will be instrumental in shaping the future of CRS management and improving outcomes for patients with cancer.

## Figures and Tables

**Table 1:** Therapeutic Agents Associated with CRS.

Drug	Indications	Mechanism of Action	CRS Incidence	Severe CRS Incidence	References
**Monoclonal Antibodies**					
Rituximab	B-cell non-Hodgkin lymphoma, chronic lymphocytic leukemia, autoimmune diseases.	Anti-CD20 monoclonal antibody	1–2% (during first infusion)	Rare (<1%)	Shimabukuro-Vornhagen et al. [1], Maloney et al. [11], Kulkarni et al. [12]
Alemtuzumab	Chronic lymphocytic leukemia, multiple sclerosis	Anti-CD52 monoclonal antibody	~10% (initial doses)	<5%	Shimabukuro-Vornhagen et al. [1]
Tocilizumab	Rheumatoid arthritis, systemic juvenile idiopathic arthritis, CRS treatment.	Anti-IL-6R monoclonal antibody	Rare for CRS (<1%).	Rare (<1%)	Schiff et al. [13]
Blinatumomab	Relapsed/refractory B-cell acute lymphoblastic leukemia (ALL).	CD19 × CD3 BiTE	~60–70%	~10–15%	Shimabukuro-Vornhagen et al. [1]
**CAR T-cell Therapies**					
Tisagenlecleucel	B-cell ALL, diffuse large B-cell lymphoma (DLBCL).	Anti-CD19 CAR T	~37–49%	15–23%	Shimabukuro-Vornhagen et al. [1], Porter et al. [14]
Axicabtagene Ciloleucel	Relapsed/refractory large B-cell lymphoma.	Anti-CD19 CAR T	~80%	~20%	Shimabukuro-Vornhagen et al. [1], Gagelmann et al. [15]
Lisocabtagene Maraleucel	Relapsed/refractory large B-cell lymphoma.	Anti-CD19 CAR T	~42–46%	2–3%	Shimabukuro-Vornhagen et al. [1], Abramson et al. [16]
Ciltacabtagene Autoleucel	Relapsed/refractory multiple myeloma.	Anti-BCMA CAR T	~95%	~5–10%	Shimabukuro-Vornhagen et al. [1], Abebe et al. [17]
**Immune Checkpoint Inhibitors**					
Nivolumab	Melanoma, non-small cell lung cancer (NSCLC), renal cell carcinoma.	Anti-PD-1 monoclonal antibody	Rare (<1%)	Very rare (<1%)	Ceschi et al. [18]
Pembrolizumab	Advanced melanoma, NSCLC, head and neck cancer, and other solid tumors.	Anti-PD-1 monoclonal antibody	Rare (<1%)	Very rare (1%)	Zhang et al. [19], Ceschi et al. [18]
Ipilimumab	Advanced melanoma, renal cell carcinoma, colorectal cancer.	Anti-CTLA-4 monoclonal antibody	Rare (2%)	Rare (1%)	Menakuru et al. [20], Ceschi et al. [18]
**Cytokines**					
Interleukin-2	Metastatic renal cell carcinoma, metastatic melanoma.	IL-2 receptor agonist	~30–50%	5–10%	Shimabukuro-Vornhagen et al. [1], Ko et al. [21], Rokade et al. [22], Davar et al. [23]
Interferons	Hepatitis C, Multiple Sclerosis, and some cancers.	Type I interferon	Low (<10%)	<1%	Denstaedt et al. [24]
**Bispecific Antibodies**					
Teclistamab	Relapsed/refractory multiple myeloma.	BCMA × CD3 BiTE	~72%	1–2%	Martin et al. [25], Hamadeh et al. [26]
Amivantamab	NSCLC with EGFR exon 20 insertion mutations.	EGFR × MET BiTE	10–15%	Rare (<1%)	Park et al. [27]
Mosunetuzumab	Relapsed or refractory B-cell non-Hodgkin lymphoma.	CD20 × CD3 BiTE	~40–50%	<5%	Bartlett et al. [28]
Cevostamab	Relapsed or refractory multiple myeloma.	FcRH5 × CD3 BiTE	~50–70%	~5–10%	van de Donk et al. [29], Pan et al. [30]
**Other Biologic Agents**					
Anakinra	Rheumatoid arthritis, cytokine-driven inflammatory diseases, CRS treatment.	IL-1 receptor antagonist	Rare (<5%)	Rare (<1%)	Gazeau et al. [31]
Efgartigimod	Myasthenia Gravis.	Neonatal Fc receptor antagonist	Rare (<10%)	<1%	Howard et al. [32]
**Small Molecule Inhibitors**					
Lenalidomide	Multiple Myeloma, Myelodysplastic syndrome, Mantle Cell lymphoma	Immunomodulatory drug (IMiD); enhances T/NK-cell function, Cereblon modulator degrading Ikaros/Aiolos TFs	Rare (<10%)	<1%	McCarthy et al. [33]
Thalidomide	Multiple Myeloma, Erythema Nodosum Leprosum.	Immunomodulatory drug (IMiD); inhibits TNF-α production, modulates cereblon, suppresses angiogenesis	Rare (<10%)	<1%	Stewart et al. [34]

**Table 2:** Infectious Causes of CRS-Like Syndromes by Kingdom.

Kingdom	Pathogen	Clinical Manifestations	Micro Related CRS-like Syndrome	References
**Bacterial**				
	*Mycobacterium tuberculosis*	Fever, hepatosplenomegaly, cytopenia, hyperferritinemia, lymphadenopathy	Hemophagocytic Lymphohistiocytosis	Kurver et al. [70]
	*Nocardia spp.*	Fever, lung nodules, brain abscesses, skin infections, systemic inflammation	Cytokine Storm Syndrome, Immune reconstitution inflammatory syndrome	Gur et al. [71]
	*Staphylococcus aureus*	High fever, hypotension, rash, multi-organ failure	Toxic Shock Syndrome	Silversides et al. [72]
	*Streptococcus pyogenes*	High fever, hypotension, rash, multiorgan failure, necrotizing fasciitis	Streptococcal TSS	Stevens et al. [73]
**Viral**				
	SARS-CoV-2	Fever, respiratory distress, shock, hyperinflammation, multiorgan failure	Cytokine Storm Syndrome, Multisystem Inflammatory Syndrome in Children	Gao et al. [74], Lee et al. [65]
	EBV	Fever, hepatosplenomegaly, lymphadenopathy, pancytopenia, hyperferritinemia	Hemophagocytic Lymphohistiocytosis, Macrophage Activation Syndrome	Marsh et al. [75], Gomez et al. [76]
	Influenza	High fever, myalgia, hypotension, respiratory distress, ARDS	Hypercytokinemia, Macrophage Activation Syndrome (MAS)	Wei et al. [77], Jayashree et al. [78]
	Dengue	Fever, hypotension, vascular leakage, thrombocytopenia, hemorrhage	Dengue Shock Syndrome, Macrophage Activation Syndrome (MAS), Hemophagocytic Lymphohistiocytosis	Rajapakse et al. [79], Ab-Rahman et al. [80]
	Ebola	Fever, hemorrhage, hypotension, multi-organ failure	Post-Ebola Syndrome Cytokine Storm	Reynard et al. [81]
	CMV	Fever, hepatosplenomegaly, lymphadenopathy, cytopenia	Hemophagocytic Lymphohistiocytosis	Kampouri et al. [53]
	HIV	Persistent fever, pancytopenia, hepatosplenomegaly, immune dysregulation	Hemophagocytic Lymphohistiocytosis	Tabaja et al. [82]
**Fungal**				
	*Aspergillus* spp.	Fever, respiratory distress, lung nodules, CNS involvement (in severe cases)	Invasive Apsergillosis IRIS	Jung et al. [83]
	*Candida* spp.	Fever, sepsis-like shock, multi-organ dysfunction in severe invasive cases	Candidemia-Associated Hyperinflammatory Response	Steinbrick et al. [84]
	*Cryptococcus* spp.	Fever, headache, altered mental status, meningitis, CNS involvement	Cryptococcal IRIS	Wiesner et al. [85]
**Parasitic**				
	*Strongyloides stercoralis*	Fever, sepsis-like shock, eosinophilia, multi-organ dysfunction	Hyperinfection Syndrome	Kassalik et al. [86]
	*Toxoplasma gondii*	Fever, headache, seizures, encephalitis, chorioretinitis	Toxoplasma encephalitis	Marra et al. [87]
	*Leishmania spp.*	Fever, hepatosplenomegaly, pancytopenia, hyperferritinemia	Hemophagocytic Lymphohistiocytosis	Neycheva et al. [88]

## Abbreviations

CRS = Cytokine Release Syndrome, SIRS = Systemic Inflammatory Response Syndrome, MAS = Macrophage Activation Syndrome, CAR = Chimeric Antigen Receptor, BiTE = Bispecific T-cell Engager, ICANS = Immune Effector Cell-Associated Neurotoxicity Syndrome, IRS = Immune Reconstitution Syndrome = IRIS = immune reconstitution inflammatory syndrome, ASTCT = American Society for Transplantation and Cellular Therapy, TNF-α = Tumor Necrosis Factor-alpha, ALL = Acute Lymphoblastic Leukemia, IFN-γ = Interferon-gamma, IL-2 = Interleukin-2, IL-8 = Interleukin-8, MIP-1β = Macrophage Inflammatory Protein-1 beta, IL-6 = Interleukin-6, IL-1 = Interleukin-1, HLH = Hemophagocytic Lymphohistiocytosis, BCMA = B-cell Maturation Antigen, CMV = Cytomegalovirus, HSV = Herpes Simplex Virus, HHV-6 = Human Herpesvirus-6, PML = Progressive Multifocal Leukoencephalopathy, ID = Infectious Disease(s), CRP = C-Reactive Protein, NfL = Neurofilament Light Chain.
